# Effect of intradialytic oral nutritional supplementation on nutritional markers in malnourished chronic hemodialysis patients: prospective randomized trial

**DOI:** 10.1186/s12882-023-03181-7

**Published:** 2023-05-04

**Authors:** Mohamed Sary Gharib, Mariem Shaker Nazeih, Tamer Wahid El Said

**Affiliations:** 1 Division of Nephrology, Department of Internal Medicine, Faculty of Medicine, Ain Shams University, Cairo, Egypt; 2 Division of Nephrology, Department of Internal Medicine, Ahmed Maher Teaching Hospital, Cairo, Egypt

**Keywords:** Protein-energy wasting, Oral nutritional supplement, Nutritional markers, Chronic hemodialysis

## Abstract

**Background and objectives:**

Malnutrition is prevalent in chronic hemodialysis (HD) patients. It increases mortality and negatively affects quality of life. This study aimed to assess the effect of intradialytic oral nutritional supplement (ONS) on nutritional markers in chronic HD patients with protein energy wasting (PEW).

**Methods:**

This 3-month prospective, open-label, randomized controlled trial included 60 chronic HD patients with PEW. The intervention group (30 patients) received intradialytic ONS and dietary counseling, whereas the control group (30 patients) received only dietary counseling. Nutritional markers were measured at the beginning and end of the study.

**Results:**

The mean age of the patients was 54 ± 12.7 years, and that of the HD vintage was 64 ± 49.3 months. Compared to the control group, the intervention group showed a significant increase in serum albumin (*p* < 0.001), prealbumin (*p* < 0.001), cholesterol (*p* = 0.016), body mass index (BMI) (*p* = 0.019), serum creatinine/body surface area (BSA) (*p* = 0.016), and composite French PEW score (*p *= 0.002), as well as a significant decrease in high-sensitivity C-reactive protein (hs-CRP) (*p *= 0.001). The total iron binding capacity, normalized protein nitrogen appearance, and hemoglobin levels increased significantly in both groups.

**Conclusion:**

Intradialytic ONS and dietary counseling for three months were more effective than dietary counseling alone in terms of improving nutritional status and inflammation in chronic HD patients, as evidenced by increases in serum albumin, prealbumin, BMI, serum creatinine/BSA, composite French PEW score, and a decrease in hs-CRP.

## Introduction

Protein-energy wasting (PEW) is a common problem in maintenance hemodialysis (MHD) patients, with a prevalence of 28–54% [1]. It is defined as the depletion of body proteins and energy reserves [2]. Reduced oral intake is the primary contributor to PEW and can result from anorexia, dietary restrictions, and difficulties in food provision and preparation [3, 4]. Other factors associated with PEW include inadequate physical activity, nutritional losses in dialysate, and endocrine and metabolic disorders, such as hyperparathyroidism, hypothyroidism, hypogonadism, growth hormone resistance, and insulin resistance [3, 4]. PEW can lead to infection, cardiovascular disease, frailty, and depression, all of which increase the risk of morbidity and mortality and decrease quality of life [3, 4].

Several diagnostic criteria have been suggested for PEW. In 2008, the ISRNM expert panel [2] recommended four categories of criteria (Table 1) for identifying PEW. At least one criterion in at least three categories should be fulfilled for a diagnosis of PEW. In 2014, Moreau-Gaudry et al. [5] developed a French PEW score (Table 1) composed of four criteria, one for each category of PEW. Each criterion is graded one if the value exceeds the threshold and zero if it is less. The final score is the sum of the criteria grades. According to this score, patients are classified as having normal nutritional status (score 4), slight wasting (score 3), moderate wasting (score 2), or severe wasting (score 0–1). The authors found that a reduced score is associated with reduced survival and its improvement is associated with better survival [5].

Various nutritional strategies have been proposed for the treatment of PEW. These include dietary counseling, administration of oral nutritional supplements (ONS), enteral tube feeding, intradialytic partial parenteral nutrition, and total parenteral nutrition [6]. Dietary counseling addressing patient-specific barriers can optimize the nutritional status of MHD patients [7]. But, without the addition of a nutritional supplement, it may take longer to achieve nutritional repletion and may not be as successful in maintaining it [8]. However, other researchers have found it ineffective when used in isolation [9–12]. Nutritional advice should focus on increasing protein and calorie consumption while minimizing the intake of phosphorus, potassium, and sodium [13, 14]. Prolonged periods of fasting, such as skipping meals due to laboratory testing, avoiding eating during dialysis, and decreasing oral intake during hospitalization and intercurrent illnesses, should be discouraged [6].

ONS should be prescribed when dietary counseling alone fails to meet the gap between spontaneous dietary consumption and recommended nutritional requirements [15]. These can be either commercial formulae [8–11, 16–20] or food-based supplements [12] that provide protein and/or energy. They can be administered either at home or during dialysis. ONS administration during HD compensates for the altered protein metabolism caused by dialysis with uncommonly reported side effects [4]. Protein turnover studies in malnourished MHD patients who received intradialytic ONS revealed a positive whole-body net balance and an improvement in skeletal muscle protein homeostasis [21]. The efficacy of ONS in improving the nutritional status in HD patients has been supported by both randomized and non-randomized clinical trials. Oral supplements improve dietary protein [10, 11, 16, 17, 19] and energy [10, 11] intake, biochemical nutritional markers, such as albumin [8–10, 16, 17, 19, 20], prealbumin [16], total protein [17], body mass [9, 10, 16, 17], fat mass [9, 10], muscle mass [10], and nutritional score [11, 12]. Improvements have also been observed in physical functioning [18], inflammation [17, 18], and quality of life [11, 12, 22]. Furthermore, observational studies have found that MHD patients who received ONS had lower hospitalization [20] and mortality rates [23].

This study aimed to assess the effects of intradialytic renal non-specific, protein- and energy-based, oral nutritional supplement (ONS) on individual nutritional markers as well as the composite PEW score in malnourished prevalent hemodialysis patients.

## Materials and methods

### Study population

This study included chronic hemodialysis patients at a single hemodialysis center. Eligible participants were adults aged ≥ 18 years who had been receiving maintenance HD thrice weekly for at least 6 months, and had PEW defined as serum albumin levels ≤ 3.5 g/dl [24–26] and prealbumin levels < 20 mg/dl [27]. Given that the serum albumin level was less than the threshold used in the French PEW score [5] (Table 1), all patients included in the study had a score of ≤ 3 (slight wasting). The exclusion criteria were infections, cirrhosis, enteropathy, metastatic malignancy, current treatment with immunosuppressive agents or corticosteroids, history of allergy to any component of the ONS used, and inadequate dialysis (Kt/V < 1.2). All patients received conventional HD with a high-flux polysulfone dialyzer and bicarbonate-containing dialysate solution. The participants signed an informed written consent before enrollment in the study. The study protocol was approved by the local ethics committee.

**Table 1:** The French PEW score in comparison with PEW criteria proposed by the ISRNM expert panel

Category	ISRNM criteria	French PEW score criteria
Biochemical		
	Serum albumin < 3.8 g/dl	Serum albumin ≤ 3.8 g/dl
	Serum prealbumin < 30 mg/dl	
	Serum cholesterol < 100 mg/dl	
Body mass		
	BMI < 23 kg/m^2^	BMI ≤ 23 kg/m^2^
	Unintentional weight loss: >5% or > 10% in 3 and 6 months, respectively	
	Body fat < 10%	
Muscle mass		
	Losses of > 5% or > 10% in 3 and 6 months, respectively	Serum creatinine/BSA ≤ 3.8 mg/dl/m^2^
	MUAMC reduction > 10% below the median of the reference population	
	Creatinine appearance	
Dietary intake		
	low DPI^a^ <0.8 g/kg/day	nPNA ≤ 0.8 g/kg/day
	low DEI < 25 kcal/kg/day	

^a^Can be assessed using a dietary diary or nPNA calculation

Abbreviations: PEW: protein energy wasting; ISRNM: international society of renal nutrition and metabolism; BMI: body mass index; MUAMC: mid upper arm muscle circumference; BSA: body surface area; DPI: dietary protein intake; DEI: dietary energy intake; nPNA: normalized protein nitrogen appearance

### Study design

This prospective three-month open-label randomized controlled trial was conducted from August 2022 to November 2022. Sixty-four eligible patients were enrolled and randomly allocated 1:1 to intervention group (IG) and control group (CG). A simple randomization table generated by a computer software program was used to determine randomization. The sequence holder, who was located off-site, determined the allocation. A nutritionist counseled both groups on increasing dietary protein intake (≥ 1.2 g/kg/day) and consuming calorie-dense foods (30–35 kcal/kg/d). In addition, patients in the IG consumed oral nutritional supplement (ONS) powder (NEO-MUNE; Otsuka, Table 2) during each HD session throughout the study period. The powder was administered in a dose of 100 g per session, mixed and dissolved in 250 ml of warm water by shaking in a closed container, and then consumed throughout the session.

Patients’ demographics and causes of kidney failure were collected from medical records. Body fluid volume and lean body weight were assessed by bioimpedance analysis using a body composition monitor (Fresenius Medical Care, Germany) after the end of the HD session on the same day as blood sampling. Dry body weight was determined as: post-dialysis weight – overhydration estimated by body composition analysis. Body mass index (BMI) was calculated as: dry body weight (kg) / height (m^2^). Body surface area (BSA, m^2^) was calculated using the Boyd formula [28]: 0.03330 x weight ^0.6157−0.0188 log10 weight^ x height ^0.3^, and spKt/Vurea was estimated using the second-generation Daugirdas formula [29]. Normalized protein nitrogen appearance (nPNA) (g/kg/day) was calculated by the formula [30]: predialysis BUN / (25.8 + (1.15 x Kt/V) + (56.4 / Kt/V)) + 0.168.

The primary outcome of this study was the change in serum albumin level. The secondary outcomes were the changes in (i) other biochemical nutritional and inflammatory markers, including serum prealbumin, cholesterol, total iron binding capacity (TIBC), high-sensitivity C-reactive protein (hs-CRP), and ferritin; (ii) dry weight and BMI as surrogates for body mass; (iii) serum creatinine/BSA and lean body weight as surrogates for muscle mass; (iv) nPNA as a surrogate for dietary protein intake; and (v) the composite French PEW score.

**Table 2:** Composition of the oral nutritional supplement (NEO-MUNE; Otsuka) Per 100 g

Element	Concentration	Element	Concentration
Energy, kcal	423	Vitamin B6, mg	1.56
Protein, g	26.07	Vitamin B12, µg	9.55
Fat, g	12.08	Folic acid, µg	215.1
Carbohydrate, g	52.58	Pantothenic acid, mg	2.55
Sodium, mg	332.5	Niacin, mg	11.10
Potassium, mg	420.7	Biotin, µg	179
Phosphorus, mg	95.02	Choline, mg	155
Magnesium, mg	118.8	Iron, mg	8.01
Chloride, mg	346.6	Zinc, mg	6
Calcium, mg	249.2	Copper, mg	0.61
Vitamin A, IU	1379	Manganese, mg	0.80
Beta-carotene, IU	1209	Iodine, µg	59.63
Vitamin D, IU	144	Carnitine, mg	39.80
Vitamin E, IU	43.40	Taurine, mg	46.01
Vitamin K1, µg	36	Chromium, µg	27.25
Vitamin C, mg	159.2	Selenium, µg	14.50
Vitamin B1, mg	1.58	Molybdenum, µg	33.50
Vitamin B2, mg	1.03		

### Laboratory parameters

The serum albumin levels were measured using the bromocresol green method. High-sensitivity CRP (hs-CRP) was measured using turbidometric immunoassay (normal range < 3.0 mg/L). Other chemical measurements, such as serum prealbumin, total cholesterol, TIBC, creatinine, urea, ferritin, calcium, phosphorus, iPTH, and hemoglobin, were performed using routine methods. Blood samples were collected before the mid-week HD sessions. Samples for serum prealbumin measurement were centrifuged, and the serum was stored at -80 °C until analysis. A post-dialysis sample was drawn for the measurement of post-dialysis urea to calculate the spKT/V.

### Statistical analysis

Statistical analysis was performed using SPSS software (version 26.0). Continuous variables were presented as mean ± standard deviation (SD). Categorical variables were presented as numbers and proportions. Comparison of baseline patients’ characteristics between groups was assessed using the Chi-square test or independent sample t-tests, as appropriate. Comparisons of patient characteristics at the start and end of the study within each group, as well as at the end of the study between groups, were assessed using mixed-design repeated measures ANOVA with Bonferroni post-hoc correction for pairwise comparisons. Differences in French PEW scores at the start and end of the study within each group were evaluated using the Wilcoxon signed-rank test, and between the two groups at month 3 using the Mann-Whitney U test. *P* value of less than 0.05 was considered statistically significant.

## Results

### Baseline demographics of participants

**Fig. 1:**
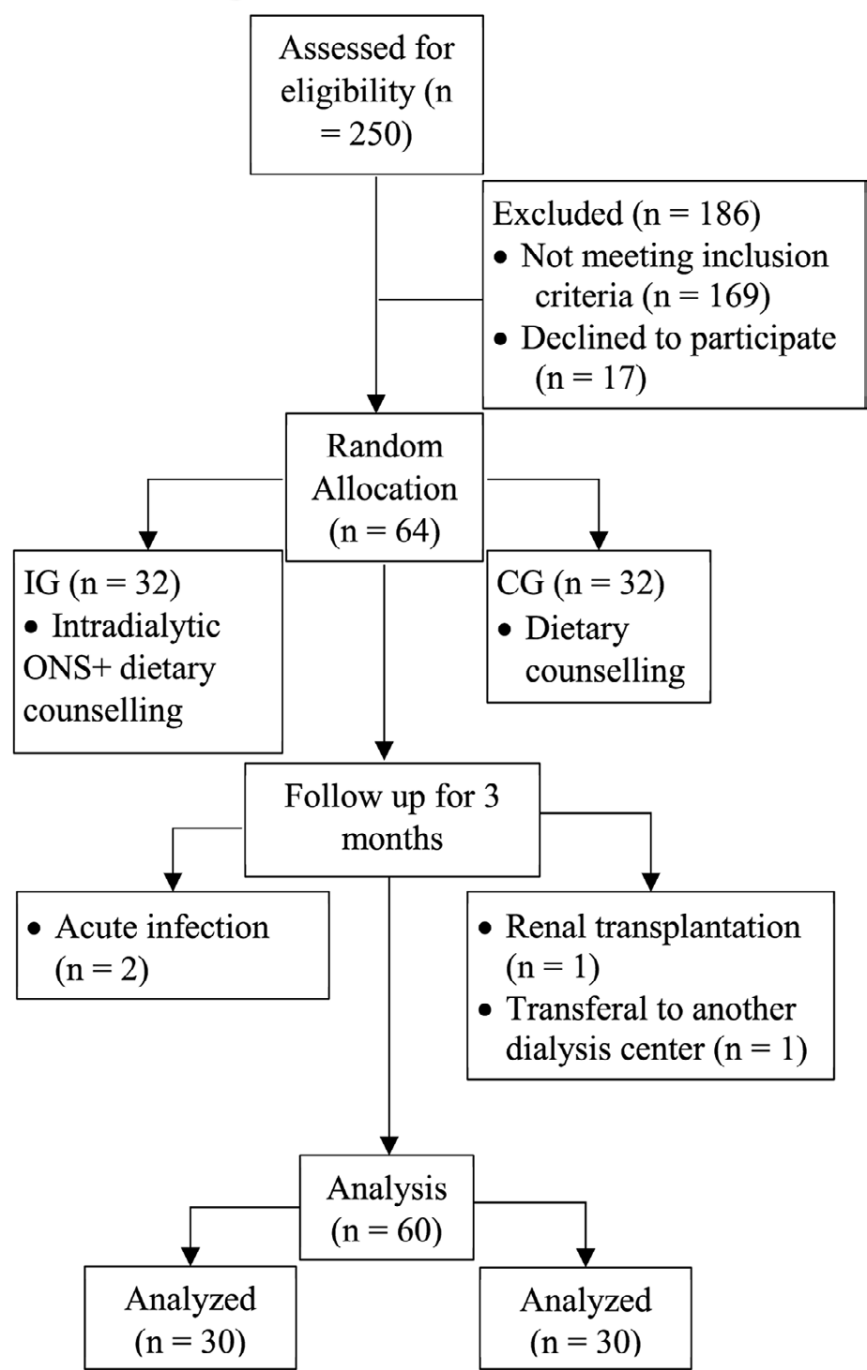
Flow diagram of the study. Abbreviations: IG: intervention group; CG: control group; ONS: oral nutritional supplement

Sixty-four patients met the inclusion criteria and were randomly allocated 1:1 in both groups. Four patients dropped out before the end of the study: two in the IG due to acute infection, two in the CG due to renal transplantation, and one transferred to another dialysis unit. Consequently, 60 patients (30 in each group) were included in the final analysis (Fig. 1). The mean age of the patients was 54 ± 12.7 years, and the mean dialysis vintage was 64 ± 49.3 months. Primary renal diseases were diabetes mellitus in 16 (26.6%), hypertensive nephrosclerosis in 16 (26.6%), chronic glomerulopathy in 6 (10%), obstructive uropathy in 2 (3.3%), ADPKD in 2 (3.3%), and unknown in 18 (30%). The baseline patients’ characteristics are shown in Table 3.

### Baseline clinical and laboratory parameters of study population

The mean serum albumin and prealbumin levels were 3 ± 0.28 g/dl and 11.7 ± 3.7 mg/dl, respectively. Patients in the CG exhibited significantly higher baseline interdialytic weight gain (IDWG) and mildly higher serum prealbumin levels, whereas patients in the IG had mildly higher nPNA. Other baseline clinical and laboratory parameters did not differ significantly between the groups. Forty-eight patients in our cohort had severe wasting (PEW score of 0–1), 11 had moderate wasting (PEW score of 2), and one patient had slight wasting (PEW score of 3). No significant differences were found between the PEW scores of the two groups at the start of the study (Table 3).

**Table 3:** Baseline characteristics of the study population

Parameter		All patients	Intervention group	Control group	*P* value
Number		60	30	30	
Age, year		54.2 ± 12.7	55.7 ± 11.73	52.8 ± 13.6	0.376
Male gender, n (%)		34(56.6)	17(28.3)	17(28.3)	1
Hemodialysis vintage, month		64.4 ± 49.3	56.7 ± 47.7	72.0 ± 50.4	0.233
Dry weight, kg		59.2 ± 8.47	59.9 ± 8.56	58.5 ± 8.47	0.530
Height, cm		168 ± 7.59	168 ± 6.56	168 ± 8.60	0.827
Body mass index, kg/m^2^		20.8 ± 2.73	21.1 ± 2.77	20.6 ± 2.70	0.429
Interdialytic weight gain, kg		2.3 ± 0.87	2.0 ± 0.92	2.7 ± 0.70	0.005
Primary kidney disease, n (%)					0.585
	Hypertension	16(26.6)	9(15)	7(11.6)	
	Diabetes mellitus	16(26.6)	9(15)	7(11.6)	
	Others/unknown	28(46.6)	12(20)	16(26.6)	
Statin use, n (%)		9(15)	6(10)	3(5)	0.236
Serum albumin, g/dl		3 ± 0.28	3 ± 0.27	3 ± 0.30	0.788
Serum prealbumin, mg/dl		11.7 ± 3.70	10.9 ± 3.65	12.6 ± 3.60	0.064
Total iron binding capacity, µg/dl		186 ± 19.7	185 ± 24.3	186 ± 14.2	0.928
Total cholesterol, mg/dl		103 ± 23.1	106 ± 27.3	99.6 ± 17.8	0.238
High-sensitivity c-reactive protein, mg/l		6.4 ± 2.41	6.58 ± 2.24	6.40 ± 2.74	0.778
Serum ferritin, ug/l		561 ± 467.8	544 ± 404	578 ± 529	0.780
Hemoglobin, g/dl		9.2 ± 1.56	9.3 ± 1.59	9.1 ± 1.56	0.696
Sodium, mEq/l		140 ± 2.54	140 ± 1.86	141 ± 3.04	0.173
Potassium, mEq/l		4.4 ± 0.64	4.3 ± 0.60	4.5 ± 0.68	0.323
Phosphorus, mg/dl		6 ± 1.43	6 ± 1.29	6 ± 1.58	0.979
Calcium, mg/dl		7.7 ± 0.90	7.7 ± 0.93	7.7 ± 0.88	0.876
Intact parathyroid hormone, pg/ml		464 ± 242	482 ± 215	446 ± 269	0.570
Pre-dialysis urea, mg/dl		146 ± 26.2	152 ± 26.8	140 ± 24.8	0.094
Post-dialysis urea, mg/dl		48.4 ± 9.03	48.8 ± 10.01	47.9 ± 8.08	0.693
Single pool Kt/V		1.3 ± 0.09	1.3 ± 0.10	1.3 ± 0.08	0.153
Creatinine, mg/dl		6.2 ± 1.25	6.3 ± 1.25	6.1 ± 1.26	0.611
Creatinine/Body surface area, mg/dl/m^2^		3.1 ± 0.64	3.1 ± 0.61	3.1 ± 0.68	0.856
nPNA, g/kg/day		0.75 ± 0.11	0.77 ± 0.11	0.72 ± 0.10	0.064
Lean body mass, kg		48.1 ± 5.74	48.5 ± 5.67	47.7 ± 5.88	0.564
French PEW score, n (%)					
0–1		48(80)	24(80)	24(80)	
2		11(18.3)	5(16.7)	6(20)	
3		1(1.7)	1(3.3)	0(0)	
4		0(0)	0(0)	0(0)	
French PEW score, median (minimum-maximum)		1(0–3)	1(0–3)	1(0–2)	0.399

Abbreviations: nPNA: normalized protein nitrogen appearance, PEW: protein energy wasting

French PEW score: 4 (normal nutritional status), 3 (slight wasting), 2 (moderate wasting), 0–1 (severe wasting)

### Comparison of individual nutritional parameters at the start and end of the study

**Fig. 2:**
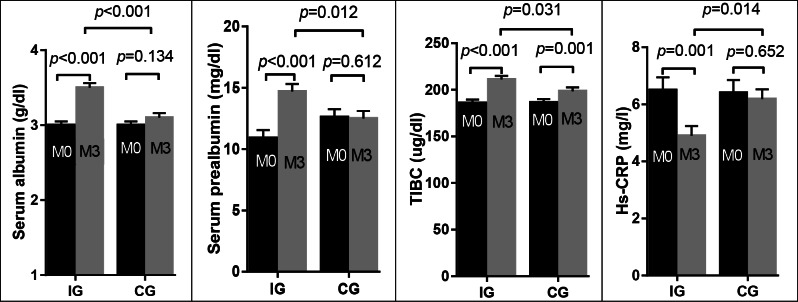
Pairwise comparison of biochemical nutritional parameters in the intervention group (IG) and the control group (CG) at baseline (M0) and the end (M3) of the study using Bonferroni post-hoc correction. Values were presented as estimated marginal means ± standard error

After 3 months of ONS use, significant increases in the following nutritional parameters were found in the IG, but not in the CG: serum albumin (*p* < 0.001 vs. *p* = 0.134), prealbumin (*p* < 0.001 vs. *p* = 0.612), total cholesterol (*p* = 0.016 vs. *p* = 0.106), dry weight (*p* = 0.022 vs. *p* = 0.211), BMI (*p* = 0.019 vs. *p* = 0.210), and serum creatinine/BSA (*p* = 0.016 vs. *p* = 0.965). This led to a significant difference in the levels of serum albumin (*p* < 0.001) and prealbumin (*p* = 0.012) between both groups at the end of the study. A significant increase in the following parameters was found in both groups: TIBC (*p* < 0.001 [IG] vs. *p* = 0.001 [CG]), nPNA (*p* < 0.001 [IG] vs. *p* < 0.001 [CG]), and hemoglobin (*p* = 0.048 [IG] vs. *p* = 0.025 [CG]). On the other hand, hs-CRP decreased significantly in the IG but not in the CG (*p* = 0.001 vs. *p* = 0.652, respectively), leading to a significant difference in hs-CRP levels (*p* = 0.014) between both groups at the end of the study. Serum ferritin levels decreased in IG (*p* < 0.001) and CG (*p* = 0.064), but the change was not statistically significant for the latter. No significant changes were observed in other parameters in either group. The changes in nutritional markers are shown in Table 4; Fig. 2.

### Comparison of composite French PEW score at the start and end of the study

In the IG, the PEW score increased in 15 patients, remained unchanged in 13, and decreased in two. The score improved by one grade in 11 patients, two grades in three patients, and three grades in one patient. In the CG, the PEW score increased in seven patients, remained unchanged in 18, and decreased in five. In six patients, the score improved by one grade, while that of one patient improved by two grades. The PEW score increased significantly in the IG compared to that in the CG (*p* = 0.002 vs. *p* = 0.564), resulting in a significant difference in the score at the end of the study (*p* = 0.001) (Table 4).

**Table 4:** Comparison of clinical and biochemical parameters at baseline and the end of the study

Parameter		Intervention group (n = 30)			Control group (n = 30)			*P value
		Start	End	*P* value	Start	End	*P* value	
Dry weight, kg		59.9 ± 1.55	60.4 ± 1.49	0.022	58.5 ± 1.55	58.8 ± 1.49	0.211	0.444
BMI, kg/m^2^		21.1 ± 0.50	21.3 ± 0.47	0.019	20.6 ± 0.50	20.7 ± 0.47	0.210	0.337
Albumin, g/dl		3.0 ± 0.05	3.5 ± 0.06	< 0.001	3.0 ± 0.05	3.1 ± 0.06	0.134	< 0.001
Prealbumin, mg/dl		10.9 ± 0.66	14.7 ± 0.61	< 0.001	12.6 ± 0.66	12.5 ± 0.61	0.612	0.012
TIBC, ug/dl		185 ± 3.64	211 ± 4.01	< 0.001	186 ± 3.64	198 ± 4.01	0.001	0.031
Hs-CRP, mg/l		6.5 ± 0.45	4.9 ± 0.34	0.001	6.4 ± 0.45	6.19 ± 0.34	0.652	0.014
Cholesterol, mg/dl		106 ± 4.22	120 ± 4.41	0.016	99.6 ± 4.22	108 ± 4.41	0.106	0.064
Ferritin, ug/l		544 ± 86.1	482 ± 78.3	< 0.001	578 ± 86.1	554 ± 78.3	0.064	0.521
Hemoglobin, g/dl		9.3 ± 0.28	9.8 ± 0.16	0.048	9.1 ± 0.29	9.7 ± 0.16	0.025	0.714
Sodium, mEq/l		140 ± 0.46	141 ± 0.46	0.265	141 ± 0.46	140 ± 0.46	0.265	0.514
Potassium, mEq/l		4.3 ± 0.11	4.4 ± 0.11	0.765	4.5 ± 0.11	4.6 ± 0.11	0.647	0.238
Phosphorus, mg/dl		6.05 ± 0.26	6.09 ± 0.25	0.895	6.06 ± 0.26	6.08 ± 0.25	0.947	0.978
Calcium, mg/dl		7.7 ± 0.16	7.8 ± 0.15	0.792	7.7 ± 0.16	7.7 ± 0.15	0.914	0.755
iPTH, pg/ml		482 ± 44.5	514 ± 34.7	0.114	446 ± 44.5	458 ± 34.7	0.554	0.259
Creatinine, mg/dl		6.30 ± 0.23	6.65 ± 0.18	0.009	6.13 ± 0.23	6.15 ± 0.18	0.887	0.063
Creatinine/Body surface area, mg/dl/m^2^		3.7 ± 0.14	3.9 ± 0.12	0.016	3.72 ± 0.14	3.73 ± 0.12	0.965	0.166
nPNA, g/kg/day		0.77 ± 0.02	0.90 ± 0.02	< 0.001	0.72 ± 0.02	0.78 ± 0.02	< 0.001	0.092
Lean body mass, kg		48.5 ± 1.05	48.7 ± 1.04	0.054	47.6 ± 1.05	47.7 ± 1.04	0.227	0.536
French PEW score, n (%)								
	0–1	24(80)	13(43.3)		24(80)	24(80)		
	2	5(16.7)	14(46.7)		6(20)	6(20)		
	3	1(3.3)	2(6.7)		0(0)	0(0)		
	4	0(0)	1(3.3)		0(0)	0(0)		
French PEW score, median (minimum-maximum)		1(0–3)	2(0–4)	0.002	1(0–2)	1(0–2)	0.564	0.001

Values were presented as estimated marginal means ± standard error

*P value for comparison between groups at the study end

Abbreviations: BMI: body mass index, TIBC: total iron binding capacity, hs-CRP: high-sensitivity C-reactive protein, iPTH: intact parathyroid hormone, BSA: body surface area, nPNA: normalized protein nitrogen appearance, PEW: protein energy wasting

French PEW score: 4 (normal nutritional status), 3 (slight wasting), 2 (moderate wasting), 0–1 (severe wasting)

## Discussion

This study investigated the effect of intradialytic ONS administration for 3 months on nutritional markers in chronic HD patients with PEW. We reported the changes in the four PEW categories. Biochemical aspect was assessed using serum albumin, prealbumin, and cholesterol levels. Body mass was assessed using BMI. Muscle mass was assessed using serum creatinine/BSA and lean body weight. Dietary intake was assessed using nPNA as a surrogate for protein intake. Additionally, we studied changes in two other chemical parameters related to malnutrition, namely TIBC as a surrogate for transferrin level, which is included in the malnutrition-inflammation score [31], and hs-CRP as a surrogate for inflammation. The findings showed a significant increase in serum albumin, prealbumin, BMI, and serum creatinine/BSA, as well as a decrease in hs-CRP in the intervention group (IG) but not in the control group (CG).

Serum albumin and prealbumin concentrations are robust markers of nutrition in HD patients [32, 33]. Epidemiological studies revealed that a 0.2–0.3 g/dl increase in serum albumin is associated with a 20% reduction in mortality after controlling for confounding factors [34]. Therefore, serum albumin was considered the strongest biochemical parameter to predict mortality in HD patients according to the updated NKF/KDOQI guideline 2020 [15]. However, a recently published position paper by the American Society for Enteral and Parenteral Nutrition recommends not using albumin and prealbumin as markers of PEW [35]. The authors claimed that these visceral proteins are indicators of inflammation and that inflammation, not these proteins, is associated with malnutrition. Inflammation modifies the priorities of hepatic protein synthesis, leading to decreased visceral proteins generation. They contended that albumin and prealbumin are associated with the risk of adverse events rather than with PEW. In our study, serum albumin and prealbumin were the only nutritional parameters in the inclusion criteria, with cut-off levels of ≤ 3.5 g/dl for serum albumin and < 20 mg/dl for serum prealbumin. To improve the specificity of these chemical markers for PEW detection, we chose thresholds lower than those recommended by the ISRNM expert panel and the French PEW score (< 3.8 g/dl for albumin and < 30 mg/dl for prealbumin).

In this study, we found a significant increase in serum albumin and prealbumin concentrations after 3 months in the IG. In agreement with these results, previous studies reported similar conclusions [9, 10, 16, 20, 36]. Sezer et al. [9] examined the effect of a renal-specific formula given to malnourished MHD patients at a dose of 28–42 g of protein and 800–1200 kcal/day for 6 months. Serum albumin levels significantly increased only in the experimental group. In a cross-over trial [10], the effects of oral administration of branched-chain amino acids (BCAA) at a dose of 12.5 g per day for six months were evaluated in elderly MHD patients. The results showed a significant increase in serum albumin levels by month 3. Cano et al. [16] reported a significant increase in serum albumin and prealbumin levels in a group of MHD patients treated for two years with ONS containing 25 g of protein and 500 kcal per day. The increase in these markers was observed at month 3 and remained until the end of the study in prealbumin and until month 18 in albumin. Cheu et al. [20] showed that MHD patients who received ONS had significantly higher serum albumin trajectories than the control group. Caglar et al. [36] investigated the effect of a 6-month intradialytic administration of renal-specific, protein- and energy-based ONS in MHD patients who had a significant decrease in body weight, albumin, prealbumin, or transferrin over the preceding 3 months. They observed a significant increase in serum albumin and prealbumin levels at the end of the study period. In contrast to our findings, Caetano et al. [37] reported no change in serum albumin levels after 6 months of intradialytic administration of an ONS containing 31 g of protein, but the control group in this study showed a significant decrease in serum albumin. Furthermore, Fouque et al. [11] found that a renal-specific ONS providing 500 kcal and 18.75 g of protein per day for three months was ineffective in increasing serum albumin and prealbumin levels in MHD patients with mild malnutrition and low protein intake. Similarly, Tomayko et al. [18] reported no change in serum albumin levels after 6 months of ONS use. These inconsistencies could be attributed to differences in patient characteristics, nutritional formulas used, associated comorbidities, and initial serum albumin and prealbumin levels. For instance, patients examined by Tomayko et al. [18] had serum albumin levels greater than those in our study (4 g/dl vs. 3.3 g/dl).

Total iron-binding capacity (TIBC), an indirect measure of transferrin, is an independent predictor of malnutrition in HD patients [38]. In this study, TIBC increased significantly in both groups but more so in the IG, and there was a significant difference between the two groups at the end of the study. Contrary to our results, previous investigators [36] reported no change in transferrin levels after six months of intradialytic ONS consumption. Again, these contradictory results could be attributed to the differences in the study populations, associated comorbidities, and ONS components. For example, the investigators of the aforementioned study used ONS, which provided 16 g of protein per dose in every dialysis session, whereas we used a supplement that provided 26 g of protein.

Dry weight and BMI were two other nutritional markers that improved in the intervention arm of this study. Cano et al. [16] found that malnourished HD patients who received protein- and energy-based ONS for two years had a significant increase in BMI. Sezer et al. [9] found that after 6 months of daily renal-specific nutritional formula, there was a significant increase in dry weight, whereas the control group showed a significant decrease in dry weight at the end of the study. Hiroshige et al. [10] observed a significant increase in dry weight in elderly HD patients who received 12 g of oral BCAA daily beginning at month 3. Fouque et al. [11] and Caglar et al. [36] reported an increasing trend in body weight in patients who received ONS for six and three months, respectively, but this was not statistically significant. In contrast, Tomayko et al. [18] did not observe a change in body weight after intradialytic oral protein supplementation in HD patients, but the study population did not meet the criteria for malnutrition.

Compared to the CG, the IG showed improvements in surrogates of muscle mass, reaching statistical significance for serum creatinine and creatinine/BSA, but not for lean body weight. Serum creatinine is affected by muscle mass and meat intake [2]. The more pronounced changes in serum creatinine and creatinine/BSA compared to lean body weight could be attributed to increased meat consumption by IG patients. In elderly MHD patients who received daily ONS, lean body weight increased significantly by month 6 [10]. In contrast, Sezer et al. [9] reported no change in muscle mass in MHD patients who received daily ONS for 6 months, but there was a significant decrease in muscle mass in the control group.

There was a significant increase in nPNA in both groups. The increase in nPNA in the control group could be attributed to increased protein consumption as a result of dietary counseling or to an increase in endogenous protein catabolism. We did not measure protein consumption by dietary recall; therefore, we were unable to clearly distinguish between the two explanations. Excess endogenous protein catabolism in the control group is a less appealing explanation because it would be associated with loss of muscle mass, which was not observed in our study. Bolasco et al. [17] reported a significant increase in nPNA after 3 months of oral amino acid supplementation at a dose of 12 g daily in MHD patients, which was not observed in the control group. Cano et al. [16] reported a significant increase in nPNA starting from month 3 in a cohort of MHD patients treated with ONS, which contained 25 g protein and 500 kcal/day for 2 years. In contrast, Fouque et al. [11] studied the effect of daily renal-specific nutritional formula administration and reported a significant increase in nPNA at months 1 and 2, but the changes after 3 months were not significant. Similarly, other researchers [9] found no increase in nPNA in MHD patients who received ONS for six months.

The significant improvement in inflammation in the IG, as demonstrated by the significant decrease in hs-CRP, is an important finding of this study. This could be secondary to the improvement in nutritional status, or inflammation could be a primary issue that the anti-inflammatory elements of the ONS could be addressed. Reduced inflammation may have contributed to the improvement of malnutrition in the second case. Consistent with our findings, Tomayko et al. [18] noticed a decrease in serum IL-6 and CRP levels in MHD patients who received soy and whey protein for 6 months during dialysis, although this was not statistically significant for the latter. Similarly, Bolasco et al. [17] reported a reduction in CRP levels in patients who received 12 g of oral amino acid supplementation daily for 3 months.

We observed a significant increase in composite PEW score in the IG, whereas no changes were observed in the CG. This is due to improvements in the grades of individual components. Fouque et al. [11] examined the effects of ONS consumption on composite subjective global assessment (SGA). They reported an increase in the median SGA classification from 4 (mild-to-moderate risk of malnutrition) to 6 (no risk of malnutrition) in HD patients receiving ONS, whereas the median classification of the control group decreased and remained at mild-to-moderate risk. Similarly, Caglar et al. [36] reported a 14% increase in the mean SGA score after three months of intradialytic administration of a nutritional formula specific for HD patients.

All patients who completed the study were compliant. No significant adverse effects of ONS were reported, particularly no increase in predialysis serum phosphate or potassium levels, in the IG. In addition, there was no reported increase in the interdialytic weight gain. Two patients in the IG experienced mild nausea during the first few days of the study. They continued the study at their own will, and nausea subsided without the need to discontinue the supplement.

One of the strengths of this study is that it was a prospective, controlled, randomized trial. The control arm allowed us to isolate the beneficial effects of ONS on nutritional parameters. Furthermore, ONS was administered to patients during dialysis sessions to ensure compliance and compensate for protein and energy losses. To the best of our knowledge, this is the first study to investigate the effect of intradialytic NEO-MUNE on nutritional markers on chronic HD patients. This study has some limitations. First, it was a single-center study with a small sample size. Second, the study was conducted over a short period, whereas clinical nutritional changes may take longer to be observed.

## Conclusions

Intradialytic NEO-MUNE administration combined with dietary counseling for 3 months for malnourished chronic HD patients was found to be more effective than dietary counseling alone in terms of improving nutritional status and inflammation, as evidenced by increases in serum albumin, prealbumin, BMI, serum creatinine/BSA, composite French PEW score, and decreased hs-CRP levels.

## Data Availability

The study’s datasets are not publicly available due to privacy concerns, but they are available from the corresponding author upon reasonable request.

## Declarations

### Study registration

The study was registered in Pan African Clinical Trial Registry (www.pactr.org) database (first registration date: 13/09/2022; registration number: PACTR202209728463176).

## Abbreviations

HD: hemodialysis
ONS: oral nutritional supplement
PEW: protein energy wasting
BMI: body mass index
IG: intervention group
CG: control group
ADPKD: autosomal dominant polycystic kidney disease
IDWG: interdialytic weight gain
ISRNM: international society of renal nutrition and metabolism
TIBC: total iron binding capacity
